# Development and Study of Novel Ultrafiltration Membranes Based on Cellulose Acetate

**DOI:** 10.3390/polym16091236

**Published:** 2024-04-28

**Authors:** Anna Kuzminova, Mariia Dmitrenko, Roman Dubovenko, Margarita Puzikova, Anna Mikulan, Alexandra Korovina, Aleksandra Koroleva, Artem Selyutin, Konstantin Semenov, Rongxin Su, Anastasia Penkova

**Affiliations:** 1 St. Petersburg State University, 7/9 Universitetskaya nab., St. Petersburg 199034, Russia; m.dmitrienko@spbu.ru (M.D.); r.dubovenko@spbu.ru (R.D.); st095099@student.spbu.ru (M.P.); annamikanna@gmail.com (A.M.); st098339@student.spbu.ru (A.K.); aleksandra.koroleva@spbu.ru (A.K.); a.selyutin@spbu.ru (A.S.); 2 Pavlov First Saint Petersburg State Medical University, L’va Tolstogo ulitsa 6-8, St. Petersburg 197022, Russia; semenov1986@yandex.ru; 3 State Key Laboratory of Chemical Engineering, School of Chemical Engineering and Technology, Tianjin University, Tianjin 300072, China; surx@tju.edu.cn

**Keywords:** cellulose acetate, carbon nanoparticles, ultrafiltration, post-treatment

## Abstract

Recently, increasing attention of researchers in the field of membrane technology has been paid to the development of membranes based on biopolymers. One of the well-proven polymers for the development of porous membranes is cellulose acetate (CA). This paper is devoted to the study of the influence of different parameters on ultrafiltration CA membrane formation and their transport properties, such as the variation in coagulation bath temperature, membrane shrinkage (post-treatment at 80 °C), introduction to casting CA solution of polymers (polyethylene glycol (PEG), polysulfone (PS), and Pluronic F127 (PL)) and carbon nanoparticles (SWCNTs, MWCNTs, GO, and C_60_). The structural and physicochemical properties of developed membranes were studied by scanning electron and atomic force microscopies, Fourier-transform infrared spectroscopy, X-ray photoelectron spectroscopy, and contact angle measurements. The transport properties of developed CA-based membranes were evaluated in ultrafiltration of bovine serum albumin (BSA), dextran 110 and PVP K-90. All developed membranes rejected 90% compounds with a molecular weight from ~270,000 g/mol. It was shown that the combination of modifications (addition of PEG, PS, PL, PS-PL, and 0.5 wt% C_60_) led to an increase in the fluxes and BSA rejection coefficients with slight decrease in the flux recovery ratio. These changes were due to an increased macrovoid number, formation of a more open porous structure and/or thinner top selective, and decreased surface roughness and hydrophobization during C_60_ modification of blend membranes. Optimal transport properties were found for CA-PEG+C_60_ (the highest water—394 L/(m^2^h) and BSA—212 L/(m^2^h) fluxes) and CA-PS+C_60_ (maximal rejection coefficient of BSA—59%) membranes.

## 1. Introduction

In recent years, there has been active development of biopolymer-based materials for the use in all membrane processes. Biopolymers are widely used for the production of membranes due to their high hydrophilicity and environmental friendliness. Among biopolymers, cellulose derivatives are often used, which are highly soluble polymers, in contrast to pure cellulose, and actively used in the processes of reverse osmosis, pervaporation, nanofiltration, and ultrafiltration [1,2,3,4,5,6,7,8,9,10]. Cellulose acetate (CA) was first used to prepare polymer membranes for the separation of aqueous media by reverse osmosis and ultrafiltration methods [11,12]. Moreover, CA is actively used in the textile industry (various fabrics, braids), in medicine (in drug delivery systems and as dressing materials), in the tobacco industry (for the manufacture of cigarette filters), in coatings and inks, in negatives and photographic film, etc. [13,14,15,16,17,18,19,20,21,22]. In addition, CA is an environmentally friendly natural biodegradable polymer, so it can potentially replace traditional synthetic ultrafiltration membranes [23]. Currently, active development of CA-based membranes is also underway for reverse osmosis [2,3], pervaporation [4], nanofiltration [5,6], and ultrafiltration [7,8,24,25,26,27,28,29,30,31,32,33]. CA is a hydrophilic polymer and, therefore, has high resistance to surface contamination. At the same time, membranes based on CA have low resistance to aggressive environments and chemical attack, including oxidation, and low mechanical strength. The development of CA-based membranes with improved transport, physicochemical, and operational characteristics is an urgent task [23]. One of the ways to improve the properties of porous membranes is their modification with additives and/or nanoparticles [30]. At the moment, there is a lack of data in the literature on membranes based on CA, into which both a polymer and nanoparticles are simultaneously introduced as modifiers.

The addition of polymers as additives into membrane matrices (namely, blending) has been proven for development of novel materials with specified and intermediate properties and economic advantages [30]. Membranes from blends often demonstrate improved permeability and/or selectivity (rejection) compared to membranes based on pristine polymers. In this study, to improve CA membrane performance, polysulfone (PS), Pluronic F127 (PL), polyethylene glycol (PEG), and a combination of PS-PL were chosen as additives. To the best of our knowledge, there is a limited number of publications in which the effect of these additives (without a combination of them) on the properties of CA membranes has been studied [28,29,30,31,32,34,35]. PS is actively used as membrane matrix and characterized by high molecular mobility of phenylene rings, strength, stiffness, and stability. However, its application in aqueous media is limited due to its hydrophobicity [30]. Thus, the membranes from blended CA and PS were earlier developed and studied [28,29,30,34,35]. The choice of PS as the additive is approved by its miscibility with CA, and good mechanical and chemical resistance. PEG is actively applied as a pore-forming agent for the preparation of ultrafiltration membranes. It was previously tested and showed its promise for CA-based membranes to increase membrane porosity and surface hydrophilization [31]. PL is a typical commercial amphiphilic copolymer containing hydrophilic and hydrophobic segments, which can affect the rate of membrane formation by the phase inversion method. It was chosen for CA-based membrane modification as a pore former due to its hydrophilic–lipophilic balance and high extractability into water to improve the permeability [32]. Thus, the application of these additives and their combination for CA-based membrane modification makes it possible to specifically change the internal porous structure, surface roughness, and balance, leading to improved transport characteristics.

Various nanoparticles are also used for CA to create mixed matrix membranes (MMMs), for example, mixed metal oxide nanoparticle–polymer composites (Fe-Al-Mn@chitosan) [27], aluminum oxide (Al_2_O_3_) and nano-zerovalent iron (nZVI) nanoparticles [36], blends of polyvinylpyrrolidone (PVP) and TiO_2_ nanoparticles [37], single-wall carbon nanotubes (SWCNTs) [38], N-(3-sulfopropyl)-N-(methacryloxyethyl)-N,N-dimethylammonium betaine (SBMA)-modified graphene oxide [39], graphene oxide (GO) [3,40], HKUST-1@GO [40], graphene oxide quantum dots (GQDs) [41], and metal–organic frameworks [5,6]. In this study, carbon nanoparticles (SWCNTs, multi-walled carbon nanotubes (MWCNTs), GO, and C_60_) were chosen as modifiers for modification of CA-based membranes. Among various fillers, carbon nanoparticles play a separate and important role due to their unique chemical structure and physicochemical characteristics such as large specific surface area, light weight, chemical stability, and good mechanical strength. Their introduction into polymer matrices leads to significant changes in the morphology and mechanical and physicochemical properties of membranes due to their functional groups and structure, as a result of which the membrane performance and resistance to fouling are improved [42,43,44,45,46,47]. The application of carbon nanoparticles as modifiers has been previously proven for membranes based on polyvinyl alcohol [42] and its blend with hydroxyethyl cellulose (HEC) [43], and sodium alginate (SA) [44] and its blend with HEC [48], polyphenylene oxide [49], polyphenylene isophthalamide [49,50,51,52], polyelectrolyte complex based on sodium carboxymethyl cellulose (CMC), and poly(diallyldimethylammonium chloride) (PDADMAC) [45], PS [46], etc.

Thus, the aim of this work was to study the effect of various preparation conditions, additives, modifiers, and combinations of them, on structural, physicochemical, and transport properties of a porous ultrafiltration CA-based membrane, and the development of ultrafiltration CA- based membranes with improved performance for water treatment. The novelty of this study lies in the simultaneous study of the influence of the (1) variation in temperature of the coagulation bath and the use of post-treatment at 80 °C; (2) introduction of additives (PEG, PS, and PL) into the CA matrix; and (3) introduction of carbon nanoparticles (SWCNTs, MWCNTs, GO, and C_60_) into the pristine CA matrix and matrix of polymer blends on structural, physicochemical, and transport properties of CA-based membranes. The structural and physicochemical properties of the developed CA-based membranes were studied by scanning electron microscopy (SEM), atomic force microscopy (AFM), Fourier-transform infrared spectroscopy (FTIR), X-ray photoelectron spectroscopy (XPS), and contact angle measurements. The transport properties of the developed CA-based membranes were evaluated in ultrafiltration of bovine serum albumin (BSA). Membranes with optimal properties were tested in the ultrafiltration of dextran 110 and polyvinylpyrrolidone (PVP) K-90.

## 2. Materials and Methods

### 2.1. Materials

Cellulose acetate (CA, Mn = 40,000 g/mol, pur.) was purchased from Alfa Laval (Copenhagen, Denmark) and used as a polymer matrix for the development of porous membranes. Amphiphilic copolymer polyethylene oxide-polypropylene oxide-polyethylene oxide (PEO-PPO-PEO) (PL, Pluronic F127, 12.6 kDa, pur.) from Sigma-Aldrich (St. Louis, MO, USA), polysulfone (PS, Mn = 55,000 g/mol, Ultrason S 6010, pur.) from BASF (Ludwigshafen, Germany), and polyethylene glycol (PEG-400, Mn = 400 g/mol, pur.) from NevaReactiv (Saint-Petersburg, Russia) were used as additives for porous CA membranes. Non-woven polyester substrate (Novatexx2430) from Freudenberg Filtration Technologies (Weinheim, Germany) was used as support for the preparation of porous CA membranes. Carbon nanoparticles such as single-walled carbon nanotubes (SWCNTs, puriss.), multi-walled carbon nanotubes (MWCNTs, puriss.), graphene oxide (GO, puriss.), and fullerene (C_60_, puriss.) from Fullerene Technologies (St. Petersburg, Russia) were applied for the modification of porous CA membranes. Bovine serum albumin (BSA, Mw = 67,000 g/mol, pur.) in the form of a 0.1 wt% solution in phosphate buffer (pH = 7.0–7.2), polyvinylpyrrolidone (PVP K-90, pur., 0.3 wt% in water), and dextran 110 (puriss., 0.3 wt% in water) from Sigma-Aldrich (St. Louis, MO, USA) were used for ultrafiltration experiments. N,N′-Dimethylacetamide (DMAc, puriss.) from Vecton (Saint-Petersburg, Russia) was used as solvent and without further purification.

### 2.2. Porous Membrane Preparation

The calculated amount of CA powder was dissolved in DMAc at ambient temperature with stirring, followed by ultrasonication. The addition of PS and PEG was carried out as follows: a calculated amount of polymer (PS and PEG) was added to the CA powder and dissolved in DMAc according to the method described above. The polymer solutions were heated to 40 °C with constant stirring when introducing PS in CA matrix. The addition of PL was carried out as follows: the calculated amount of PL powder was introduced into the solution of CA or CA-PS in DMAc and intensively stirred, followed by ultrasonication. Table 1 shows the compositions of polymer solutions per 100 g of solution.

Carbon nanoparticles were introduced into the polymer matrix as follows: the calculated quantity of nanoparticles was dissolved in DMAc using ultrasound. After that, CA powder or a blend of polymer powders was introduced into the resulting dispersion and dissolved according to the procedure described above. The concentration of introduced nanoparticles was 0.5 wt% by weight of the polymer or mixture of polymers.

Porous membranes were prepared by the phase inversion method: the polymer solution was applied onto non-woven polyester support (which was fixed on glass), and cast with a casting blade using a die (gap width 200 μm) and immersed in a molding bath with water [53]. Various temperatures (10, 25, and 45 °C) of the coagulation bath were used. Additionally, a method of post-treatment of porous membranes for shrinkage at elevated temperatures was as follows: at one hour after preparing, the porous membrane was immersed in water at 80 °C for 10 min [3].

### 2.3. Atomic Force Microscopy

Atomic force microscopy (AFM) was used to study the surface topography of the porous CA-based membranes. An NT-MDT NTegra Maximus atomic force microscope (NT-MDT Spectrum Instruments, Moscow, Russia) was applied to carry out the experiment under the conditions described in [54,55].

### 2.4. Scanning Electron Microscopy

Scanning electron microscopy (SEM) was used to study the cross-sectional morphology of the porous CA-based membranes. A Zeiss AURIGA Laser (Carl Zeiss SMT, Oberhochen, Germany) was applied to carry out the experiment. Cross-sections of the membranes were obtained under the conditions described in [10,53].

### 2.5. Contact Angle Measurements

The contact angle measurements were used to study the changes in surface hydrophilic/hydrophobic balance of the porous CA-based membranes. A goniometer LK-1 instrument (NPK Open Science Ltd., Krasnogorsk, Russia) was used to carry out the experiment. The DropShape software (version 1) was used to analyze the contact angle data. The contact angles were studied by the attached bubble method under the conditions described in [47].

### 2.6. Ultrafiltration Experiment

**Figure 1:**
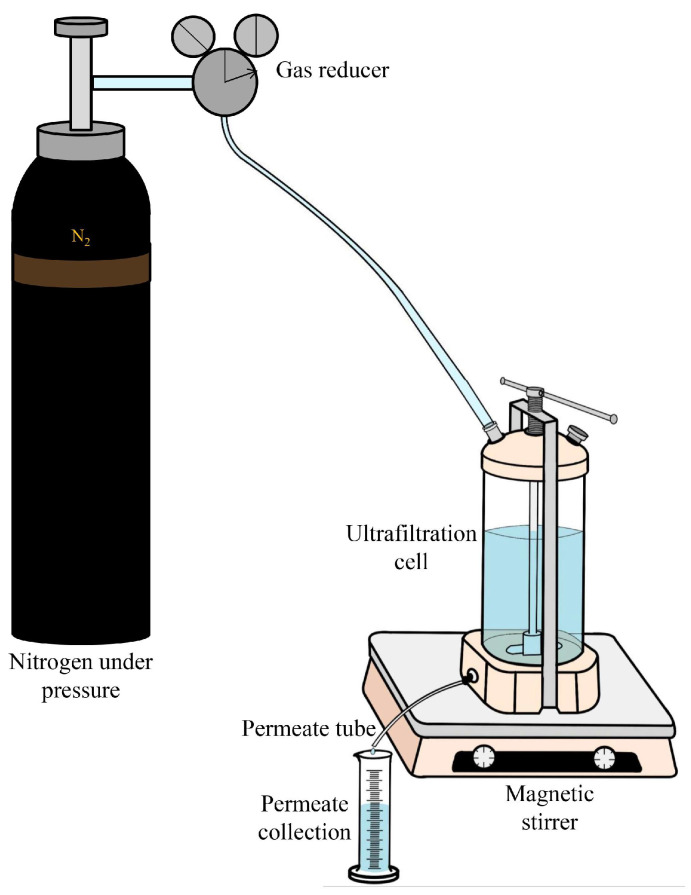
A scheme of the ultrafiltration setup.

The transport properties of the developed porous CA-based membranes were studied in an ultrafiltration experiment using a dead-end laboratory cell (NPK BIOTEST, Kirishi, Russia) in a stationary mode [47,56]. A scheme of the ultrafiltration experiment setup is presented in Figure 1. A BSA solution in phosphate buffer (0.1 wt% at pH = 7.0–7.2), PVP K-90 (0.3 wt%), and dextran 110 (0.3 wt%) were used as feeds for ultrafiltration separation.

To calculate the pure water and feed flux of the CA-based membranes, the following equation was used [57]:

(1) $$J=\frac{V}{A\cdot t},$$

where *V* (L) is the permeate volume, *A* is the effective area of the membrane, and *t* is the time of the measurement (h).

The content of BSA in the permeate and feed was studied by spectrophotometry using a PE-5400UV spectrophotometer (ECROSKHIM Co., Ltd., Moscow, Russia) at a 280 nm wavelength. The content of PVP K-90 and dextran 110 in the permeate and feed was studied by an ITR-20 interferometer (Joint Stock Company “Zagorsky Optical-Mechanical Plant”, Sergiev Posad, Russia) at 21–24 °C. To calculate the rejection coefficient of the CA-based membranes, the following equation was used:

(2) R=1−CpCf·100%,

where *C_p_* and *C_f_* are the content of solute in the permeate and feed (wt%), respectively.

To calculate the flux recovery ratio (FRR) of the CA-based membranes, the following equation was used [58]:

(3) FRR=JJ0·100%

where *J* is the pure water flux after the feed permeation through the membrane, and *J*_0_ is the initial pure water flux.

All the data were collected 3 times, and the average values were used. The obtained average accuracies were as follows: ±0.5% for the rejection coefficient and ±5% for flux of the CA-based membranes.

## 3. Results and Discussion

### 3.1. Transport Properties

**Figure 2:**
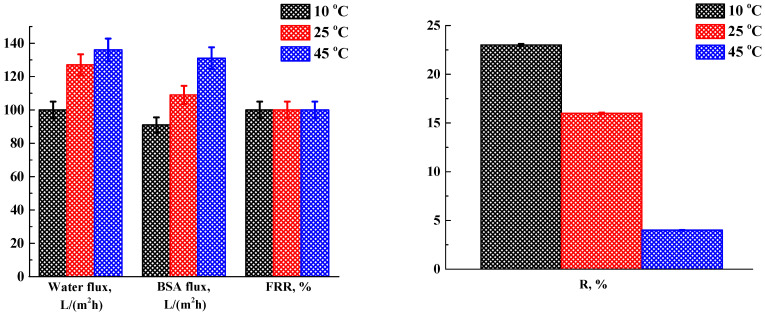
Transport characteristics of membranes based on CA, prepared at different temperatures of the coagulation bath.

In this work, the influence of the temperature of the coagulation bath on the properties of porous membranes was studied. Membranes from CA solution were prepared at different temperatures of a coagulation bath with water (10, 25, 45 °C). The transport properties of the membranes were studied during the ultrafiltration of water and BSA solution (Figure 2).

It was found that an increase in the temperature of the coagulation bath led to an increase in the water and BSA fluxes, as well as a decrease in the rejection coefficients. The increase in water and BSA fluxes may be associated with a change in the porous structure of the membranes, which can be explained as follows: with an increase in the temperature of the coagulation bath, the diffusion rate of solvent (DMAc) and nonsolvent (water) molecules increases at the interface, which leads to an increase in the deposition rate. This effect leads to a decrease in the thickness of the selective layer of porous membranes, resulting in a decrease in membrane resistance and an increase in flux. This effect was also noted earlier for membranes based on polyethersulfone (PES) [59]. The decrease in BSA rejection coefficients with increasing temperature of the coagulation bath may be associated with an increase in pore size of porous membranes. This dependency was also noted during the development of CA and PES membranes [59,60]. It was also noted that CA-based membranes, prepared at different temperatures of the coagulation bath, had good antifouling properties: 100% FRR in ultrafiltration of the BSA solution. The optimal temperature of 25 °C for the coagulation bath was chosen due to the compromise of transport parameters for the CA membrane prepared at this temperature.

**Figure 3:**
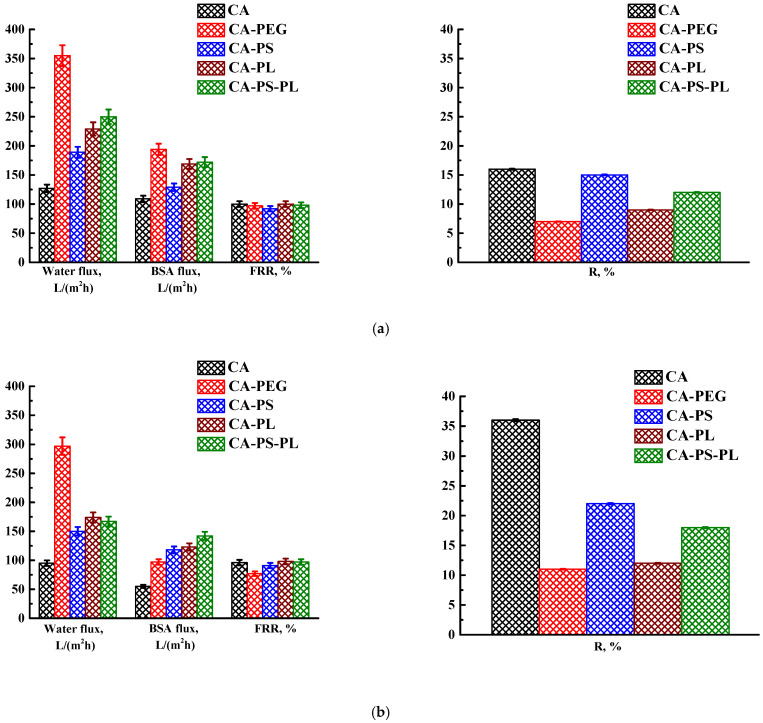
Transport characteristics of membranes based on CA and its blend with polymers prepared (**a**) without post-treatment and (**b**) with post-treatment at 80 °C.

To change transport properties, various polymers were introduced into the CA matrix (PEG, PS, PL) as additives. The membranes were formed in the coagulation bath at 25 °C followed by post-treatment at 80 °C. The transport properties of the membranes without/with post-treatment were studied during the ultrafiltration of water and BSA solution (Figure 3).

It was found that the introduction of additives (PEG, PS, PL) led to an increase in the water and BSA fluxes. The greatest increase in water flux from 127 L/(m^2^h) for the CA membrane to 355 L/(m^2^h) for the CA-PEG membrane prepared without post-treatment was noted. This CA-PEG membrane without post-treatment was also characterized by maximum 194 L/(m^2^h) flux for BSA solution, and a slightly decreased rejection coefficient (from 16 to 7%) and flux recovery ratio (from 100 to 97%) compared to the CA membrane without post-treatment. The increase in flux may be due to the fact that PEG is a hydrophilic additive. Its presence in the casting solution can accelerate the diffusion of water and facilitate the process of phase separation during the formation of the porous structure of the membrane. The decrease in BSA rejection coefficients may be due to an increase in the porosity and pore size of the membranes when PEG is washed out from the casting solution [33]. The flux recovery ratio depends on the hydrophilicity of the selective layer. The introduction of PEG into the CA matrix led to an increase in membrane hydrophilicity, causing a decrease in the FRR [56].

The introduction of PS into the CA matrix also led to an increase in the water and BSA fluxes and a decrease in the rejection coefficient and flux recovery ratio. This may be due to the fact that PS promotes the formation of larger aggregate pores (macrovoids) in the resulting porous membranes. An increase in flux upon introduction of PS into the CA matrix was also previously noted in the study of Sivakumar et al. [30]. The introduction of PL into the CA matrix also led to an increase in the water and BSA fluxes and a decrease in the rejection coefficient without a change in the flux recovery ratio values. An increase in flux upon introduction of PL into the CA matrix was also previously noted in the study of Lv et al. [32]. The simultaneous introduction of both PS and PL additives led to an increase in the water flux to 250 L/(m^2^h) and BSA flux to 172 L/(m^2^h), and a slight decrease in R to 12% and the FRR to 98%, compared to the CA membrane. The post-treatment of membranes at 80 °C resulted in a slight decrease in water and BSA fluxes and FRR, but an increase in R for membranes. These changes occur due to shrinkage of the porous structure of the membranes. This post-treatment method for shrinking the porous structure and increasing the degree of retention has been previously studied [3,61,62]. For further modification, membranes post-treated at 80 °C were used due to increased rejection ability compared to membranes without post-treatment.

**Figure 4:**
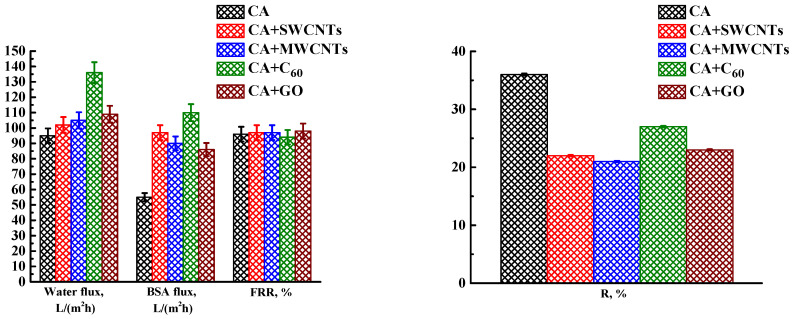
The transport properties of the CA membranes modified with carbon nanoparticles, prepared with post-treatment at 80 °C.

To assess the effect of modification with carbon nanoparticles and select the optimal modifier, CA membranes modified with 0.5 wt% various carbon nanoparticles (SWCNTs, MWCNTs, GO, C_60_) were studied. The membranes were formed in a coagulation bath at 25 °C with post-treatment at 80 °C. The transport properties of modified CA-based membranes in ultrafiltration of water and BSA solution are presented in Figure 4.

It was found that the introduction of carbon nanoparticles led to an increase in the water and BSA fluxes and a slight decrease in the rejection coefficients. The flux recovery ratio for modified membranes practically did not change compared to the pristine CA membrane. The changes in transport properties were associated with the change in the porous structure of the modified membranes (confirmed by SEM below). The introduction of carbon nanoparticles led to an increase in the size of “vacuole-like” macrovoids and a decrease in the thickness of the upper selective layer (confirmed by SEM below ), causing increased fluxes with a slight decrease in BSA rejection. Additionally, the increase in water and BSA fluxes may be associated with a slight increase in surface roughness (confirmed by AFM below), which leads to an increase in the effective surface area of the membrane and in the adsorption of water molecules on the surface. Thus, it becomes more hydrophilic, which helps to increase the rate of penetration of water molecules through the membrane [63]. For CA membranes modified with SWCNTs, MWCNTs, and GO, a slight increase in the FRR compared to the CA membrane was noted, which could be due to surface hydrophilization (confirmed by contact angle data below). The CA membrane modified with fullerene demonstrated a slight decrease in the FRR, which was associated with hydrophobization of the membrane surface (confirmed by contact angle data below) [56]. Optimal transport properties were noted for the membrane modified with fullerene (CA+C_60_ membrane): improved fluxes (136 L/(m^2^h) for water and 110 L/(m^2^h) for BSA solution) and rejection of BSA (27%), as well as a high FRR (94%).

**Figure 5:**
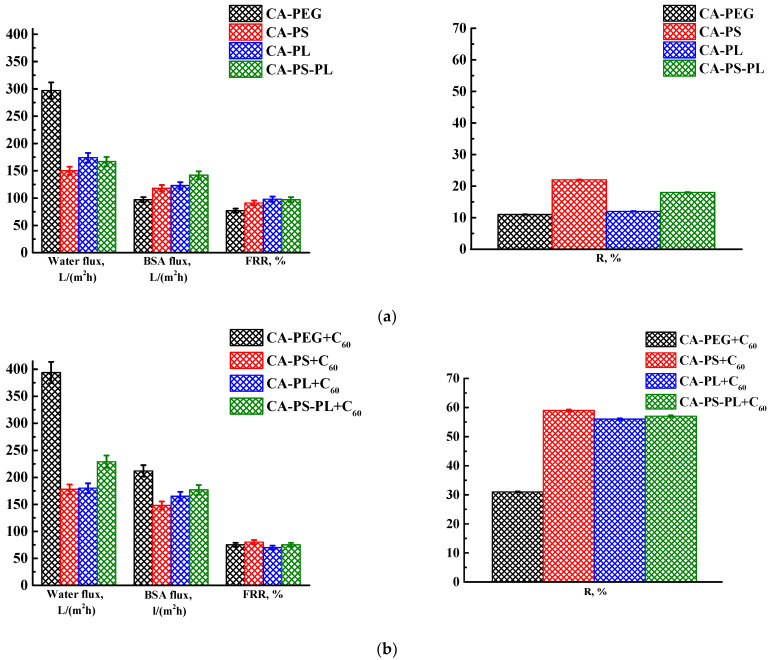
The transport properties of the CA-based membranes modified with (**a**) additives and (**b**) additives and fullerene prepared with post-treatment at 80 °C.

Porous membranes from CA with both additives and optimal modifier C_60_ (0.5 wt% related to CA or a blend of polymers) were developed using post-treatment at 80 °C. The transport characteristics of membranes were studied in the ultrafiltration of water and the BSA solution (Figure 5). The data for membranes with additives are also presented for comparison.

It was found that the introduction of fullerene into the polymer blend matrix led to an increase in the water and BSA fluxes and rejection coefficients, but with a slight decrease in the FRR. The increase in the water and BSA fluxes was associated with the change in the porous structure of the modified membranes (confirmed by SEM below). The introduction of fullerene led to an increase in the size of the “vacuole-like” macrovoids for the CA-PS+C_60_ and CA-PL+C_60_ membranes compared to unmodified polymer blended membranes (confirmed by SEM below), resulting to an increase in permeability. The introduction of fullerene into the CA-PEG and CA-PS-PL matrices led to a decrease in the selective layer thickness (confirmed by SEM below), which also caused an increase in fluxes. The decreased FRR of fullerene-modified membranes could be explained by stronger sorption of BSA in the membrane surface and pores because of membrane surface hydrophobization during C_60_ modification (confirmed by contact angle data below) [56]. This also possibly caused surface contamination (contaminant molecules become stuck in membrane pores), resulting in increased protein rejection for modified membranes. Optimal transport properties were noted for two membranes: CA-PEG+C_60_ and CA-PS+C_60_. The CA-PEG+C_60_ membrane had maximum water and BSA fluxes, while the CA-PS+C_60_ membrane had the maximum rejection coefficient of BSA.

The pristine CA-based membrane and membranes with optimal transport properties, CA-PEG+C_60_ and CA-PS+C_60_, were studied by Fourier-transform infrared spectroscopy (Section “S1 Fourier-transform infrared spectroscopy” in Supplementary Materials) and X-ray photoelectron spectroscopy (Section “S2 X-ray photoelectron spectroscopy” in Supplementary Materials). It was shown that the introduction of polymers (PEG and PS) led to a change in the structure of the polymer matrix. Additionally, the presence of fullerene in the polymer matrix was also proven using XPS analysis. These changes in the chemical composition of the membrane made it possible to obtain new characteristics of the developed membranes.

To evaluate the retention capacity of the membranes with optimal transport properties (CA-PEG+C_60_ and CA-PS+C_60_), additionally, the rejection of calibrants with different molecular weights and nature was studied. For membranes with the lowest (CA-PEG+C_60_ membrane) and highest (CA-PS+C_60_ membrane) BSA rejection coefficient, additional ultrafiltration of dextran-110 (110,000 g/mol) and PVP K-90 (360,000 g/mol) solutions was carried out. The values of the rejection coefficients of all calibrants are presented in the Table 2.

It was found that the rejection coefficients increased wise the rise in calibrant molecular weight. The rejection coefficients are lower for the CA-PEG+C_60_ membrane than for the CA-PS+C_60_ membrane. The molecular weight of the calibrants rejected at 90% was approximately calculated: ~340,000 g/mol for CA-PEG+C_60_ membrane and ~270,000 g/mol for CA-PS+C_60_ membrane.

### 3.2. Comparison of the Performance with CA-Based Membranes

The comparison of the ultrafiltration performance of the developed CA-PEG+C_60_ and CA-PS+C_60_ membranes with optimal performance, to the previous research in the CA-based membranes described in the literature for the ultrafiltration of BSA solution under close experimental conditions, is presented in Table 3.

Based on the literature review, it was shown that the developed porous CA-PEG+C_60_ and CA-PS+C_60_ membranes had higher pure water fluxes and lower BSA rejection coefficients compared to most CA-based membranes described in the literature. The presented data confirm that there is a rejection (selectivity)–permeability trade-off for a large number of porous ultrafiltration membranes: as permeability increases, the rejection coefficient decreases, and vice versa [70]. The combination of modification (additives of PEG and PS, and fullerene as a modifier) led to a more open porous structure with a thinner top selective layer and increased surface roughness (confirmed by SEM and AFM data below). It resulted in a significant increase in the permeability and decreased FRR values (due to greater membrane contamination) of the modified membranes compared to the pristine CA membrane. However, it is worth noting that for the CA-PEG+C_60_ membrane, the BSA rejection remained at a similar level compared to the CA membrane due to the preservation of the surface porous structure and hydrophilic–hydrophobic balance. By comparison, for the other CA-PS+C_60_ membrane, it increased (from 36 to 59%) due to surface hydrophobization during fullerene modification (confirmed by contact angle data below), which caused stronger protein adsorption on the surface and its improvement in retention. The lower BSA rejection compared to the literature-described CA-based membranes is also explained by the higher molecular weight of the calibrants rejected at 90% for the developed membranes (~340,000 g/mol for the CA-PEG+C_60_ membrane and ~270,000 g/mol for the CA-PS+C_60_ membrane). Thus, the developed membranes will be more promising for the industrial separation of proteins with higher molecular weights, and their high permeability will increase the productivity of the water treatment process.

### 3.3. Structure and Physicochemical Properties

To explain the obtained dependences of the transport characteristics of porous CA membranes and its composites with polymers and carbon nanoparticles, their structural features and physicochemical properties were studied by various methods. The structure and physicochemical properties of the developed porous membranes prepared with post-treatment at 80 °C were studied by SEM and AFM methods, and the attached bubble method for measuring of contact angles. Table 4 shows SEM micrographs of cross-sections at different magnifications of porous CA membranes modified with carbon nanoparticles, where red bars show the selective layer of membranes.

The introduction of carbon nanoparticles leads to the formation of an almost identical porous structure of the selective layer of the membranes (Table 4), which is reflected in similar values of the contact angle (confirmed by contact angle data below). For the unmodified CA membrane, a selective layer thickness of ~10.2 µm was noted. During modification with carbon nanoparticles, a decrease in the thickness of the top selective layer (to ~5.6 µm for CA+SWCNTs, ~9.9 µm for CA+MWCNTs, ~4.4 µm for CA+C_60_, and ~3.8 µm for CA+GO) was noted; also, macrovoids formed closer to the membrane surface, resulting in an increase in fluxes and a slight decrease in the BSA rejection coefficients (Figure 4). For all modified membranes, the vacuole-like macrovoids become slightly larger compared to the unmodified CA membrane (Table 4), and a more “open structure” is formed, which also caused an increase in membrane permeability (increase in water and BSA fluxes, Figure 4). The smallest thickness of the top selective layer was observed for membranes modified with fullerene (~4.4 µm) and GO (~3.8 µm). However, the combination of the increased size of the vacuole-like macrovoids and the decreased thickness of the top selective layer in the case of modification with fullerene led to the maximum values of water and BSA fluxes (Figure 4). Table 5 shows SEM micrographs of cross-sections at different magnifications of porous CA-based membranes modified with additives and fullerene; red bars show the selective layer of membranes.

The introduction of polymers as additives into the CA matrix leads to a change in the porous structure of the membranes (Table 5). SEM micrographs clearly reflect the dependence of the water and BSA fluxes on the porous structure. The introduction of PEG leads to the formation of the most open porous structure with a thin top selective layer (~5.3 µm). Due to the fact that during the formation of membranes a large amount of PEG is washed into the coagulation bath, and since PEG molecules are larger than water, it leads to an increase in the number of membrane macrovoids and a decrease in the selective layer thickness. Therefore, maximum values of water and BSA fluxes were observed for this membrane among all (Figure 5). The introduction of fullerene leads to a slight decrease in the size of the macrovoids, but to an increase in their number and derease in the thickness of the top selective layer (~3.2 µm), causing an increase in the water and BSA fluxes, and BSA rejection compared to the CA-PEG membrane (Figure 5). The introduction of PS into the CA matrix significantly changes the structure compared to other membranes. Pronounced oval-shaped macrovoids were not observed. A thin top selective layer (~1.9 µm) and a dense porous system of macrovoids are formed during the formation. This can be explained by the fact that PS is not washed out into the coagulation bath compared to PEG and remains in the CA matrix; its presence can be seen both in the FTIR (Figure S1 in the Supplementary Materials [5,71,72,73]) and XPS data (Figure S2 in the Supplementary Materials). PS contains both hydrophilic and hydrophobic parts, which is why its introduction so greatly changes the properties of CA-based membranes. The additional introduction of fullerene significantly changes the structure of the membrane; the decreased top selective layer (~1.3 µm) and elongated macrovoids in a more open system are formed, which lead to increased water and BSA fluxes. On the cross-section of CA-PS-based membranes, there are rounded inclusions, which are more pronounced during modification with fullerene. The CA-PL membrane is characterized by the formation of a dense structure of the top selective layer (~9.6 µm) and a small number of oval-shaped macrovoids, forming a low-permeable porous structure. The introduction of fullerene significantly loosens macrovoids and decreases the selective layer (~8.4 µm), leading to an increase in water and BSA fluxes compared to the CA-PL membrane (Figure 5). The simultaneous introduction of PS and PL leads to the formation of a thinner loose top layer (~3.2 µm), with more formed round inclusions compared to membranes modified separately with PS and PL. Further introduction of fullerene into this matrix forms a more closed porous structure with formation of a larger macrovoid number and thinner selective layer (~2.5 µm), causing the increased water and BSA fluxes of the modified membrane (Figure 5).

**Figure 6:**
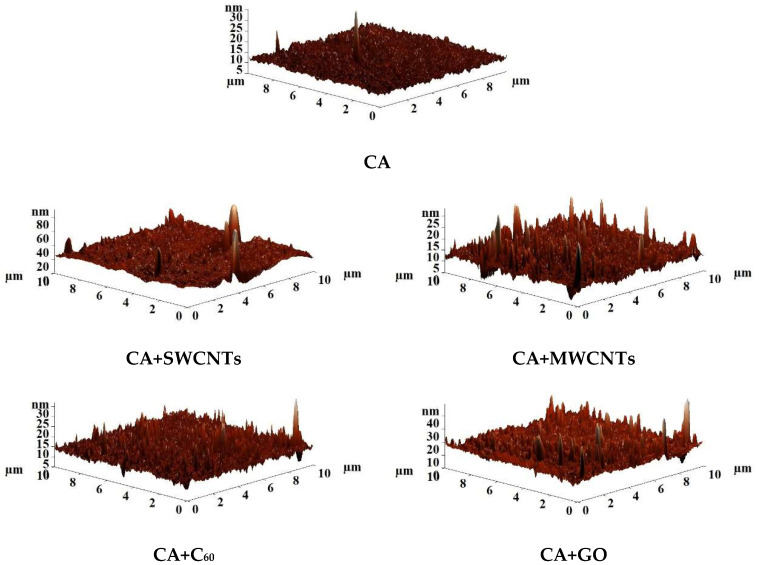
AFM images of the surface of porous CA membranes modified with carbon nanoparticles prepared with post-treatment at 80 °C.

The surface topography of the developed porous membranes was studied by the atomic force microscopy (AFM) method. Figure 6 shows AFM images of the surface of porous CA membranes modified with carbon nanoparticles.

Based on the AFM images presented in Figure 6, the average (Ra) and root mean square (Rq) surface roughness values of membranes were calculated. The values of Ra, Rq, and contact angles are presented in Table 6.

It was found that the introduction of carbon nanoparticles led to a slight increase in surface roughness, resulting in an increase in water and BSA fluxes of modified membranes. The rise in the ultrafiltration membrane surface roughness leads to an increase in the adsorption of water molecules on the surface. Thus, it becomes more hydrophilic, which helps to increase the rate of penetration of water molecules through the membrane [56]. The contact angle remained virtually unchanged during the modification of CA with carbon nanoparticles. This may be explained by the similar porous structure of the selective membrane layer (Table 3) and similar roughness values (Table 6). For CA membranes modified with SWCNTs, MWCNTs, and GO, a decrease in the contact angle values was noted, which led to a slight increase in the FRR parameter of these membranes (Figure 4). On the other hand, for CA+C_60_ membrane, an increase in contact angle was observed, resulting in greater affinity for the protein (more strongly adsorbed on the membrane surface) and causing a slight decrease in the FRR (Figure 4) [56].

**Figure 7:**
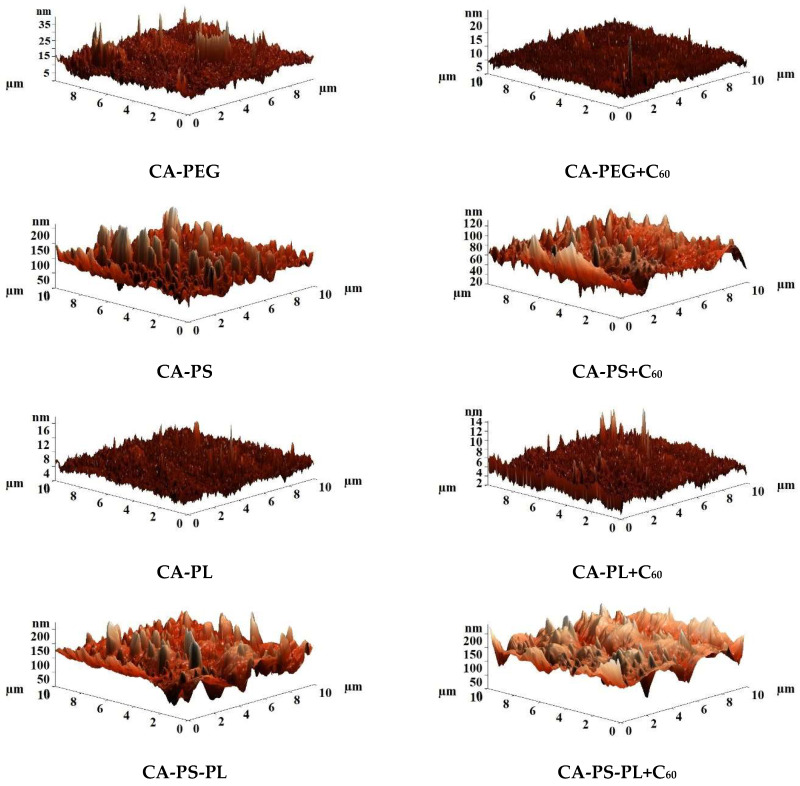
AFM images of the surface of the CA-based membranes modified with additives and fullerene prepared with post-treatment at 80 °C.

Figure 7 shows AFM images of the surface of the CA-based membranes modified with additives and fullerene with post-treatment at 80 °C.

Based on the AFM images presented in Figure 7, the average (Ra) and root mean square (Rq) surface roughness of membranes were also calculated. The values of Ra, Rq, and contact angles are presented in Table 7.

It was found that modification of CA with polymers (PEG, PS, and PL) led to an increase in surface roughness compared to the pristine CA membrane (Table 6). The rise in membrane roughness during modification leads to an increase in the adsorption of water molecules on the membrane surface, causing an increased flux [56]. The introduction of polysulfone (the CA-PS and CA-PS-PL membranes) leads to the maximum change in the surface roughness compared to the pristine CA membrane. This may be due to the fact that PS remains in the CA matrix, compared to PEG and PL as additives, which are washed into the coagulation bath during the phase inversion process. The introduction of fullerene into the matrix based on CA-PEG, CA-PL, and CA-PS-PL blends leads to a slight decrease in the surface roughness for modified membranes. The introduction of fullerene into the CA-PS matrix leads to decreased surface roughness to a larger extent. This “smoothing” of the surface during modification can also be seen in SEM micrographs (Table 5).

The introduction of additives (PEG, PS, and PL) in the CA matrix led to a slight change in contact angles; this is due to the fact that the main polymer material is CA and the additions of other polymers are insignificant for a stronger change in the contact angle. The introduction of PEG, PL, and PS-PL in the CA matrix led to a slight decrease in the contact angle compared with the pristine CA membrane (Table 6). The introduction of hydrophilic polymers into the CA matrix led to hydrophilization of the surface, which was also previously noted [32]. The introduction of PS into the CA matrix does not lead to a change in the contact angle value. The slight increase in contact angle values (surface hydrophobization) was observed for membranes modified with fullerene. Hydrophobization of the membrane surface may cause increased BSA rejection compared to unmodified membranes. The introduction of fullerene into a blend polymer matrix, as in the case of a membrane without polymer additives, leads to a slight increase in contact angles.

## 4. Conclusions

Novel ultrafiltration CA-based membranes with improved transport and antifouling properties were developed for water treatment. The change in properties of porous CA membranes was achieved by (1) variation in the temperature of the coagulation bath and the use of post-treatment at 80 °C; (2) introduction of polymers (PEG, PS, and PL) as additives into the CA matrix; and (3) introduction of carbon nanoparticles (SWCNTs, MWCNTs, GO, and C_60_) into the pristine CA matrix and matrix of polymer blends. The transport properties of membranes were evaluated in the ultrafiltration of water and BSA solution.

An increase in the temperature of the coagulation bath (10, 25, and 45 °C) led to membranes’ increased fluxes and decreased BSA rejection coefficients, while maintaining the same FRR values due to the changes in the porous structure. The optimal temperature was chosen to be 25 °C due to the compromise in the transport parameters for the CA membrane. The further modification by additives (PEG, PS, PL, and PS-PL) led to an increase in fluxes and a slight decrease in the BSA rejection and FRR values as compared with the pristine CA membrane. The introduction of 0.5 wt% carbon nanoparticles (SWCNTs, MWCNTs, GO, and C_60_) to the CA membrane led to an increase in fluxes and a slight decrease in the BSA rejection, while maintaining a similar level of FRR values. Fullerene was chosen as the optimal modifier due to the highest fluxes of the modified membrane. Under the use of combined modification (addition of PEG, PS, PL, PS-PL, and 0.5 wt% fullerene), an increase in the fluxes and BSA rejection coefficients with a slight decrease in the FRR were achieved. These obtained results were due to changes in hydrophilic–hydrophobic surface balance, roughness, selective layer thickness, porosity, and pore size.

The CA-PEG+C_60_ and CA-PS+C_60_ membranes were optimal due to the highest fluxes of 394 and 212 L/(m^2^h) and 59% maximum BSA rejection coefficient, respectively, and were tested additionally in the ultrafiltration of calibrants (dextran-110 and PVP K-90) with different molecular weights and natures. It was found that the molecular weight of the calibrants rejected at 90% was ~340,000 g/mol for the CA-PEG+C_60_ membrane and ~270,000 g/mol for the CA-PS+C_60_ membrane. Thus, based on a performance comparison with the literature-described CA-based membranes, the developed membranes are proposed to increase the industrial productivity of the water treatment process and to separate proteins with high molecular weights.

## Figures and Tables

**Table 1:** The compositions of polymer solutions per 100 g of solution.

Polymer Solution	Composition per 100 g of Solution				
	CA, g	PEG, g	PS, g	PL, g	DMAc, g
CA	17	-	-	-	83
CA-PEG	11.9	5.1	-	-	83
CA-PS	14.45	-	2.55	-	83
CA-PL	17	-	-	1.275	83
CA-PS-PL	14.45	-	2.55	1.275	83

**Table 2:** The values of the rejection coefficients of BSA, dextran-110, and PVP K-90 for the CA-PEG+C_60_ and CA-PS+C_60_ membranes.

Membrane	Rejection Coefficient, %		
	BSA (67,000 g/mol)	Dextran-110 (110,000 g/mol)	PVP K-90 (360,000 g/mol)
CA-PEG+C_60_	11	44	94
CA-PS+C_60_	59	70	100

**Table 3:** Ultrafiltration performance of the developed membranes and literature-described CA-based membranes applied for BSA rejection.

Membranes	Solute, Its Concentration and Media	Pure Water Flux, L/(m^2^h)	R, %	FRR, %	Reference
CA-PEG+C_60_	BSA (0.1 wt%) in phosphate buffer pH 7.0–7.2	394	31	75	This study
CA-PS+C_60_		178	59	80	This study
CA(85%)-PS(15%)+PVP(5%)	BSA (0.1 wt%) in phosphate buffer 0.05 M, pH 7.2	~85	~80	-	[30]
CA+PL F127 (8 wt%)	BSA (0.1 wt%) in phosphate buffer pH 7.0	~20	77	~100	[32]
CA+PEG-600 (5 wt%)	BSA (0.1 wt%)	81.85	73.5	-	[33]
Zwitterionic cellulose acetate	BSA (0.1 wt%) in phosphate buffer pH 7.4	138	99	91	[64]
CA		59	94	75	
CA+poly(ε-decalactone) (PDL)-grafted cellulose copolymer (3 wt%)	BSA (0.1 wt%) in phosphate buffer solution	13.5	-	95.6	[65]
CA+ HKUST-1@ignocellulose nanofibrils (0.55 wt%)	BSA (0.1 wt%)	207	~93	91	[66]
CA+ zinc oxide (ZnO)@graphitic carbon nitride (g-C_3_N_4_) nanocomposite (0.25 wt%)	BSA (0.025 wt%)	51.3	97.2	94.8	[67]
CA/carboxymethyl cellulose acetate (80/20 wt%)+PEG 600 (2.5 wt%)	BSA (0.1 wt%)	73.2	86.3	78.2	[68]
CA+PEG-600 (6.25 wt%)	BSA (0.1 wt%) in phosphate buffer 0.5 M, pH 7.2	98.7	~66	-	[69]

**Table 4:** SEM micrographs of cross-sections at different magnifications of porous CA membranes modified with carbon nanoparticles with post-treatment at 80 °C (red bars show the selective layer of membranes).

Membrane	2800×	5000×	9500×
CA	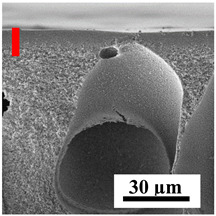	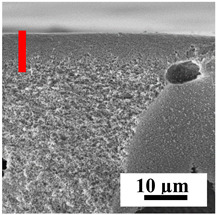	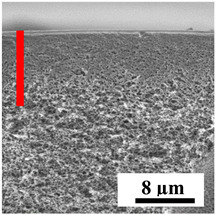
CA+SWCNTs	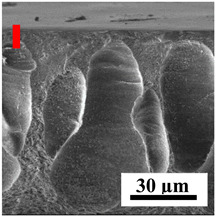	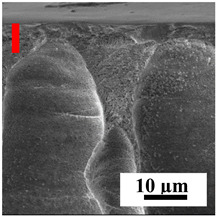	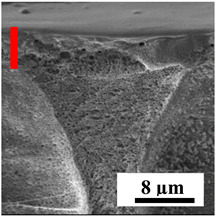
CA+MWCNTs	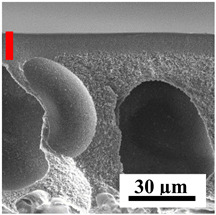	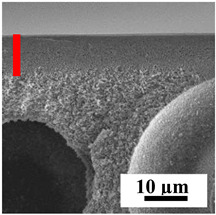	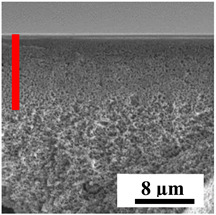
CA+C_60_	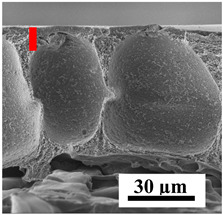	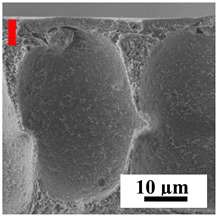	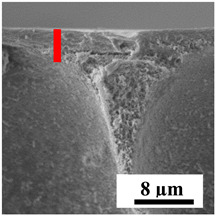
CA+GO	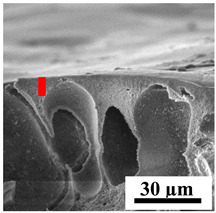	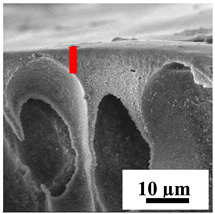	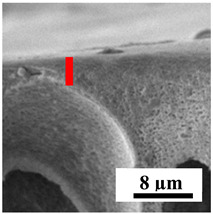

**Table 5:** SEM micrographs of cross-sections at different magnifications of porous CA-based membranes modified with additives and fullerene with post-treatment at 80 °C (red bars show the selective layer of membranes).

Membrane	2800×	5000×	9500×
CA-PEG	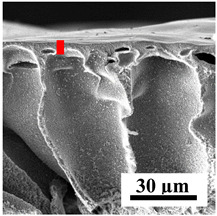	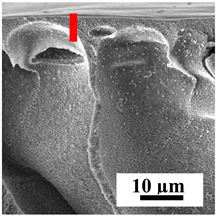	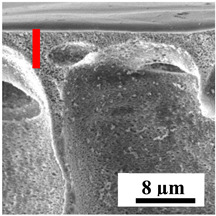
CA-PEG+C_60_	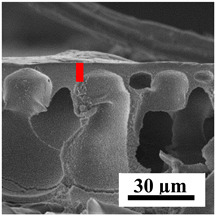	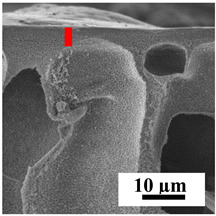	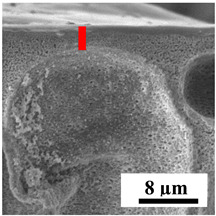
CA-PS	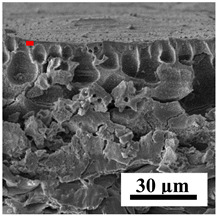	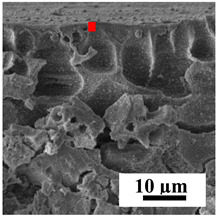	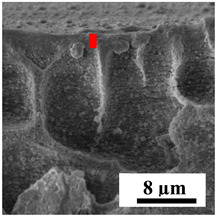
CA-PS+C_60_	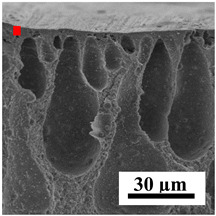	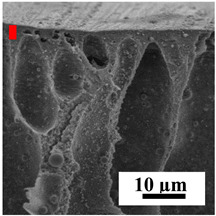	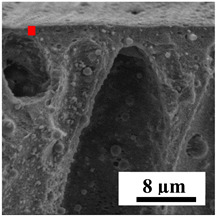
CA-PL	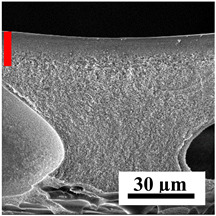	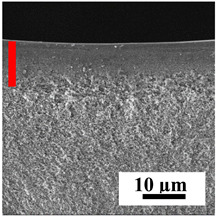	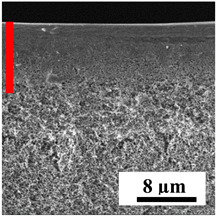
CA-PL+C_60_	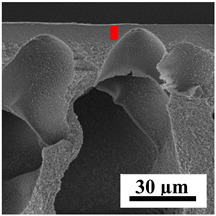	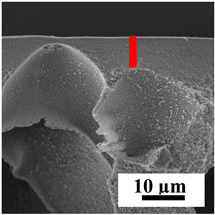	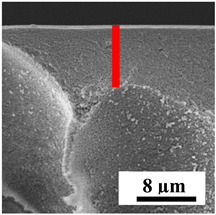
CA-PS-PL	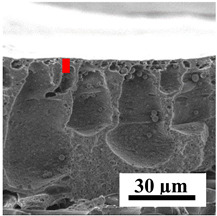	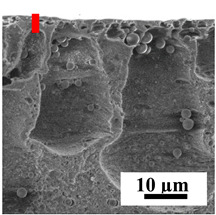	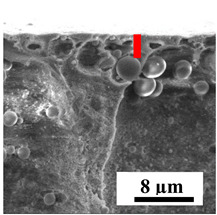
CA-PS-PL+C_60_	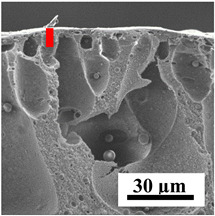	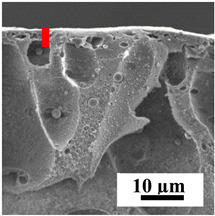	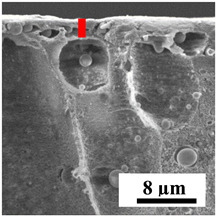

**Table 6:** The average (Ra), root-mean-square (Rq) surface roughness and contact angles for CA membranes modified with carbon nanoparticles prepared with post-treatment at 80 °C.

Membrane	Ra, nm	Rq, nm	Contact Angle, °
CA	0.8 ± 0.1	1.1 ± 0.1	41 ± 1
CA+SWCNTs	2.3 ± 0.1	4.8 ± 0.1	38 ± 1
CA+MWCNTs	1.1 ± 0.1	1.8 ± 0.1	39 ± 1
CA+C_60_	1.0 ± 0.1	1.6 ± 0.1	45 ± 1
CA+GO	1.6 ± 0.1	2.7 ± 0.1	37 ± 1

**Table 7:** The average (Ra) and root mean square (Rq) surface roughness, and contact angles, for the CA-based membranes modified with additives and fullerene prepared with post-treatment at 80 °C.

Membrane	Ra, nm	Rq, nm	Contact Angle, °
CA-PEG	1.9 ± 0.1	2.9 ± 0.1	39 ± 1
CA-PEG+C_60_	1.1 ± 0.1	1.4 ± 0.1	41 ± 1
CA-PS	16.2 ± 0.2	22.9 ± 0.2	42 ± 1
CA-PS+C_60_	10.9 ± 0.2	14.0 ± 0.2	44 ± 1
CA-PL	0.8 ± 0.1	1.1 ± 0.1	36 ± 1
CA-PL+C_60_	0.6 ± 0.1	0.9 ± 0.1	40 ± 1
CA-PS-PL	21.1 ± 0.2	27.2 ± 0.2	37 ± 1
CA-PS-PL+C_60_	18.8 ± 0.2	25.5 ± 0.2	43 ± 1

## Data Availability

The original contributions presented in the study are included in the article and supplementary material, further inquiries can be directed to the corresponding authors.

## Supplementary Materials

The following supporting information can be downloaded at: https://www.mdpi.com/article/10.3390/polym16091236/s1, Figure S1. FTIR spectra of CA, CA-PEG+C_60_ and CA-PS+C_60_ membranes; Figure S2. XPS spectra recorded from the selective membrane surface (a) survey spectrum, (b) fitted carbon C1s spectrum, (c) fitted oxygen O1s spectrum.
