# Supplementary material for: Development and Study of Novel Ultrafiltration Membranes Based on Cellulose Acetate
Source: Polymers (Basel). 2024 Apr 28;16(9):1236. doi: 10.3390/polym16091236 (PMC11085473; doi:10.3390/polym16091236)
Supplement: Supplementary file 1 [file polymers-16-01236-s001.zip › polymers-2971133-supplementary.pdf]

polymers

Supplementary Information

Development and study of novel ultrafiltration membranes based on cellulose acetate

Anna Kuzminova [1,*], Mariia Dmitrenko [1], Roman Dubovenko [1], Margarita Puzikova [1], Anna Mikulan [1], Alexandra Korovina [1], Aleksandra Koroleva [1], Artem Selyutin[1], Konstantin Semenov [2], Rongxin Su [3] and Anastasia Penkova [1,*]

[1] St. Petersburg State University, 7/9 Universitetskaya nab., St. Petersburg 199034, Russia; m.dmitrienko@spbu.ru (M.D.), r.dubovenko@spbu.ru (R.D.), st095099@student.spbu.ru (M.P.), annamikanna@gmail.com (A.M.), st098339@student.spbu.ru (Al.K.), aleksandra.koroleva@spbu.ru (A.Kor.), a.selyutin@spbu.ru (A.S.)
[2] Pavlov First Saint Petersburg State Medical University, L'va Tolstogo ulitsa 6-8, Saint Petersburg 197022, Russia; semenov1986@yandex.ru (K.S.)
[3] State Key Laboratory of Chemical Engineering, School of Chemical Engineering and Technology, Tianjin University, Tianjin 300072, PR China; surx@tju.edu.cn (R.S.)

* Correspondence: a.kuzminova@spbu.ru (A.K.), a.penkova@spbu.ru (A.P.); Tel.: +7(812)363-60-00 (ext. 3367) (A.K. and A.P.)

Citation: Kuzminova, A.; Dmitrenko, M.; Dubovenko, R.; Puzikova, M.; Mikulan, A.; Korovina, A.; Koroleva, A.; Selyutin, A.; Semenov, K.; Su, R.; et al. Development and Study of Novel Ultrafiltration Membranes Based on Cellulose Acetate. *Polymers* **2024**, *16*, 1236. https://doi.org/10.3390/polym16091236

Academic Editor: Alfredo Cassano

Received: 2 April 2024
Revised: 22 April 2024
Accepted: 25 April 2024
Published: 28 April 2024

Copyright: © 2024 by the authors. Licensee MDPI, Basel, Switzerland. This article is an open access article distributed under the terms and conditions of the Creative Commons Attribution (CC BY) license (https://creativecommons.org/licenses/by/4.0/).

Membranes with optimal transport properties (CA-PEG+C_{60} and CA-PS+C_{60}) and pristine CA membrane were studied by Fourier-transform infrared spectroscopy (FTIR) and X-ray photoelectron spectroscopy (XPS) to study of structural changes.

S1. Fourier-transform infrared spectroscopy

A BRUKER-TENSOR 27 Spectrometer (Bruker, Ettlingen, Germany) with an attenuated total reflectance (ATR) accessory was used to determine the structure of membranes in the range 600–4000 cm^{-1} at ambient temperature by Fourier-transform infrared spectroscopy (FTIR). FTIR spectra of CA, CA-PEG+C_{60} and CA-PS+C_{60} membranes were presented in Figure S1.

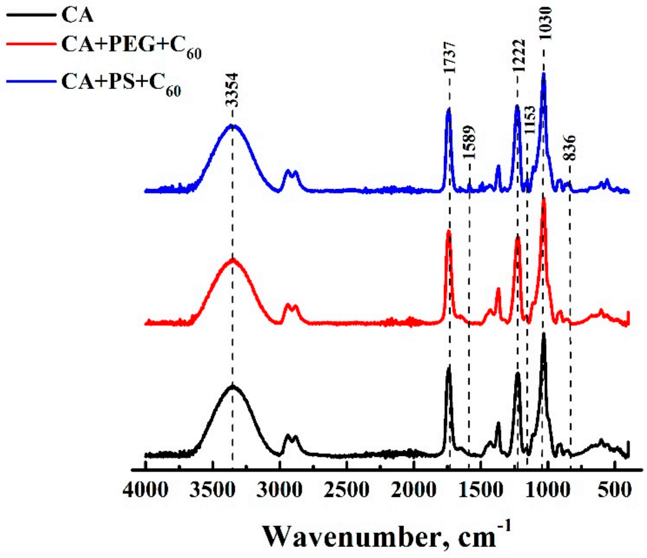

Figure S1. FTIR spectra of CA, CA-PEG+C_{60} and CA-PS+C_{60} membranes.

In the FTIR spectrum for CA membrane (Figure S1), a broad peak at 3354 cm^{-1} is a characteristic peak associated with vibrations of -OH group. The absorption at 1737 cm^{-1} is associated with stretching of C=O groups, the peaks at 1222 and 1030 cm^{-1} are related to

aromatic ring and with the –C-O- bond in the CH_2-OH group, respectively [71–73]. The introduction of PEG in the CA matrix practically did not change the FTIR spectra due to the washing out of PEG as a pore former into the coagulation bath during membrane formation. The introduction of PS in the CA leads to the appearance of new characteristic peaks of PS: peak at 1589 cm^{-1} associated with C-C bond in the aromatic, at 1153 cm^{-1} associated with stretching of –O-S-O- group, and at 836 cm^{-1} associated with C-H stretching of aromatic ring of PS [74]. The introduction of C_{60} in the polymer matrix did not change the FTIR spectra, since its peaks are overlapped by the polymer matrix due to the small addition of fullerene.

S2. X-ray photoelectron spectroscopy

Escalab 250Xi photoelectron spectrometer (Thermo Fisher Scientific, Waltham, USA) with an AlKα monochromatic radiation source (photon energy 1486.6 eV) was used to determine the structure of membranes. The XPS spectra were recorded in the constant transmission energy mode at 20 (for C1s and O1s) or 50 eV (for S2p) with an XPS spot size of 650 μm. The total energy resolution of the experiment is about 0.8 eV. A combined ion-electronic charge compensation system was used to remove the sample charge. Binding energy determining error is about 0.3 eV. The studies were carried out at room temperature in an ultrahigh vacuum of the order of $1*10^{-9}$ mbar.

To analyze the elemental and chemical composition of the selective surface of the membranes, survey (Figure S2a) and detailed carbon C1s (Figure S2b) and oxygen O1s (Figure S2c) spectra were recorded.

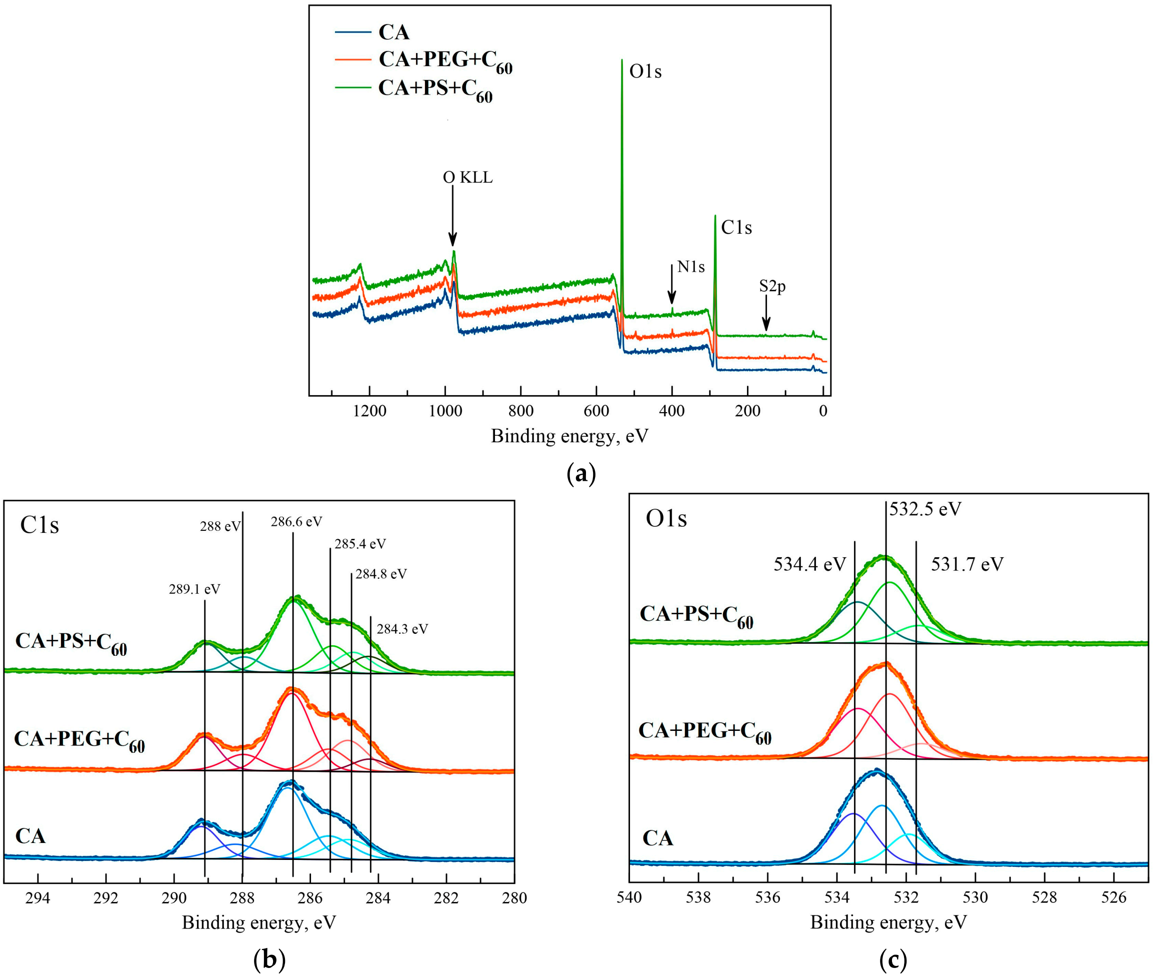

Figure S2. XPS spectra recorded from the selective membrane surface (**a**) survey spectrum, (**b**) fitted carbon C1s spectrum, (**c**) fitted oxygen O1s spectrum.

The survey spectra (Figure S2a) show that the membranes mainly contain carbon, oxygen and nitrogen. For the modified membranes, a sulfur peak was also noted, which for the membrane modified with PEG was insignificant, and for the membrane modified with PS it was larger, which corresponds to the sulfur atom of the modifier. The Figures S2b and S2c show detailed spectra of carbon (C1s) and oxygen (O1s), separated into different states for chemical analysis. The characteristic states of carbon were identified in the spectrum of the unmodified CA membrane. Since a small amount of adventitious carbon on the surface of organic substances, their states were not taken into account in the decomposition. Carbon and nitrogen compounds were also not taken into account in the decompositions, since the nitrogen content is quite low. Peaks with a binding energy of 284.4±0.3 eV, related to fullerene, are identified in the spectra of the modified (CA-PEG+C_{60} and CA-PS+C_{60}) membranes. Also, in the spectra of the modified membranes, there was a redistribution of the ratio of carbon peaks with energies of 284.8 eV and 285.4 eV, corresponding to the linear and cyclic structures, respectively. In CA-PEG+C_{60} membrane modified with polyethylene glycol, an increase in the content of the carbon state with an energy of 284.8±0.3 is observed. At the same time, in the CA-PS+C_{60} membrane modified with PS the intensity of the carbon peak with energy of 285.4±0.3 eV increased. Also for the modified CA-PEG+C_{60} and CA-PS+C_{60} membranes a peak is visible at 284.3±0.3 eV, which corresponds to fullerene in the membrane structure. At the same time, the oxygen spectra (Figure S2c) for all three membranes do not differ so significantly, since the oxygen content in the modifiers (PEG and PS) is lower, then carbon contain.

References

71. Dmitrenko, M.; Kuzminova, A.; Zolotarev, A.; Selyutin, A.; Ermakov, S.; Penkova, A. Nanofiltration Mixed Matrix Membranes from Cellulose Modified with Zn-Based Metal–Organic Frameworks for the Enhanced Water Treatment from Heavy Metal Ions. *Polymers (Basel).* **2023**, *15*, 1341, doi:10.3390/polym15061341.
72. Alhalili, Z.; Romdhani, C.; Chemingui, H.; Smiri, M. Removal of dithioterethiol (DTT) from water by membranes of cellulose acetate (AC) and AC doped ZnO and TiO2 nanoparticles. *J. Saudi Chem. Soc.* **2021**, *25*, 101282, doi:10.1016/j.jscs.2021.101282.
73. Wongsasulak, S.; Patapeejumruswong, M.; Weiss, J.; Supaphol, P.; Yoovidhya, T. Electrospinning of food-grade nanofibers from cellulose acetate and egg albumen blends. *J. Food Eng.* **2010**, *98*, 370–376, doi:10.1016/j.jfoodeng.2010.01.014.
74. Singh, K.; Devi, S.; Bajaj, H.C.; Ingole, P.; Choudhari, J.; Bhrambhatt, H. Optical Resolution of Racemic Mixtures of Amino Acids through Nanofiltration Membrane Process. *Sep. Sci. Technol.* **2014**, *49*, 2630–2641, doi:10.1080/01496395.2014.911023.

Disclaimer/Publisher's Note: The statements, opinions and data contained in all publications are solely those of the individual author(s) and contributor(s) and not of MDPI and/or the editor(s). MDPI and/or the editor(s) disclaim responsibility for any injury to people or property resulting from any ideas, methods, instructions or products referred to in the content.
